# Activated Tryptophan-Kynurenine metabolic system in the human brain is associated with learned fear

**DOI:** 10.3389/fnmol.2023.1217090

**Published:** 2023-07-28

**Authors:** Maria Rita Battaglia, Chiara Di Fazio, Simone Battaglia

**Affiliations:** ^1^Istituto di Ricovero e Cura a Carattere Scientifico Azienda Ospedaliero-Universitaria di Bologna, Policlinico S. Orsola, Bologna, Italy; ^2^Department of Psychology, Center for Studies and Research in Cognitive Neuroscience, University of Bologna, Bologna, Italy; ^3^Department of Psychology, University of Turin, Turin, Italy

**Keywords:** Tryptophan-Kynurenine pathway, learned fear, fear conditioning, PFC-dependent cognition, neuropsychiatric disorders, KYN pathway, prefrontal cortex (PFC)

## 1. What is fear learning?

Fear is widely recognized as a defensive emotion that has developed as a consequence of its adaptive function in protecting the entire animal kingdom from danger, ensuring the survival (Bouton, 2002; Milad and Quirk, 2002). Although fear associated with specific stimuli is an innate emotion (e.g., a loud noise triggering fear in infants, fear of potential predators), it can also be acquired rapidly and permanently to enable an appropriate and adaptive response to new or unpredictable environmental situations (LeDoux, 2000; Beckers et al., 2013). In an experimental context, fear learning involves a type of learning where a previously neutral stimulus is repeatedly paired with an aversive stimulus, eliciting a fear response (Maren, 2001; Bouton, 2002; Milad and Quirk, 2002; Schiller et al., 2008; Lonsdorf et al., 2017). Fear learning is recognized as the ideal experimental paradigm for investigating the anatomical, cellular, molecular, and behavioral foundations of fear learning and memory in mammals and their brains, as well as for understanding the neurobiological model of fear-related disorders in humans (Craske et al., 2006; Hartley et al., 2011; Milad and Quirk, 2012; Vervliet et al., 2013; Sevenster et al., 2015; Borgomaneri et al., 2021a; Di Gregorio et al., 2022a; Ippolito et al., 2022; Battaglia et al., 2023a). Our understanding of the fundamental neural circuitry and the cellular-molecular mechanisms underlying fear learning has significantly advanced in recent decades. In fact, our knowledge of the key brain areas involved in fear learning has greatly benefited from parallel lines of research, at least in humans (Lonsdorf et al., 2014; Dunsmoor et al., 2019).

The investigation of the molecular mechanisms involved in fear learning and in fear-related disorders, including stress-induced trauma disorders such as post-traumatic stress disorder (PTSD), is crucial for understanding the pathophysiology of these psychiatric diseases. Preclinical studies conducted on animal models have revealed the involvement of neurohormones in cognitive and emotional functions, contributing to a better understanding of important aspects of neuropsychiatric symptoms through translational research (Tanaka and Telegdy, 2008; Tanaka et al., 2011, 2012, 2022; Palotai et al., 2014; Tanaka and Vécsei, 2022). In this context, the amino acid tryptophan (Trp) is considered one of the primary contributors to stress-related diseases. The kynurenine (KYN) metabolic pathway, as a crucial component of Trp catabolism, is primarily responsible for disrupting Trp metabolism. Consequently, all processes associated with the metabolism of Trp into the KYN pathway have been extensively investigated and documented in both humans and animals (Chess et al., 2009; Balogh et al., 2021; Tanaka et al., 2021b; Polyák et al., 2023; Tajti et al., 2023). Indeed, it has been widely described that stress-induced situations can activate the hypothalamic-pituitary-adrenal (HPA) axis, leading to increased levels of corticosterone (CORT) and inflammatory processes. This, in turn, enhances the conversion of Trp to the key metabolite KYN through the activation of tryptophan 2,3-dioxygenase and indoleamine 2,3-dioxygenases (IDOs), respectively (Tanaka et al., 2021a). This diversion of Trp metabolism away from the methoxyindole metabolic pathway, which is responsible for serotonin synthesis, establishes a close link between stress and the pathophysiology of neurological and psychiatric disorders, including depression (Iaccarino et al., 2013; Tanaka et al., 2021c). Consequently, alterations in Trp metabolism may influence the development of anxiety and abnormal fear responses, as hyperfunction of KYN metabolism contributes to microglia activation in the amygdala, hippocampus, and prefrontal cortex (PFC) (Klausing et al., 2020). These regions play a crucial role in the acquisition and extinction of fear-associated memories.

## 2. Neurobiological pathway of human learned fear

A substantial body of evidence from lesion, pharmacological, and neurophysiological studies supports the notion that the amygdala plays a central role in regulating fear learning and fear extinction in humans (LeDoux, 2000). Throughout the past century, the amygdala has been recognized as “the locus of fear” (Kim and Jung, 2006): anatomically, the central nucleus of the amygdala receives sensory inputs from downstream brain areas, such as the thalamus (LeDoux et al., 1988), and projects to various autonomic, cortical, and subcortical regions involved in specific fear responses, including the prefrontal cortex (PFC), insula, and hippocampus (LeDoux, 1996; Maren and Quirk, 2004; Kim and Jung, 2006; Lonsdorf et al., 2014).

The prefrontal cortex (PFC) has been widely recognized for its critical and influential role in human fear learning, particularly in modulating the expression of learned fear in both directions. Neurophysiological studies have demonstrated the activation of the dorsomedial PFC in the long-term storage and retrieval of old fear memories (Dixsaut and Gräff, 2021), and recent research has also suggested potentially distinct contributions of the anterior and posterior subregions of the ventromedial PFC (vmPFC) to affective processes (Fullana et al., 2016; Harrison et al., 2017; Battaglia et al., 2020, 2021, 2023b). Although the prevailing understanding of vmPFC function assumes its support for successful fear extinction, it has been proposed that it may also play a significant role in fear acquisition, particularly within its posterior subregion. Specifically, the anterior vmPFC is believed to be involved in assessing the value or significance of safety signals, as evidenced by increased activity in response to safety stimuli (Phelps et al., 2004; Myers-Schulz and Koenigs, 2012; Di Gregorio et al., 2019, 2022b; Dixsaut and Gräff, 2021). On the other hand, increased activation in the posterior vmPFC (BA11) has been observed during the late stages of fear learning, highlighting the crucial role of the mid-posterior vmPFC in fear acquisition (Fullana et al., 2016; Harrison et al., 2017; Battaglia et al., 2020, 2021; Tashjian et al., 2021).

Multiple research studies and meta-analyses have consistently identified the human insula as a central region where sensory input, autonomic control, and afferents from brain regions involved in emotion processing converge (Gogolla, 2017). Functional imaging studies conducted on both rats and humans have demonstrated that the insula exhibits co-activation with a group of brain regions collectively referred to as the “fear network” (Sehlmeyer et al., 2009), including the amygdala and hypothalamus. Laboratory studies utilizing fear learning paradigms have revealed fear-induced activation of the insular cortex in both rats and humans, indicating the essential role of this region in the consolidation of learned fear and the acquisition of safety cues that suppress the expression of conditioned fear (Greco and Liberzon, 2016).

Finally, the hippocampus is believed to be involved in specific types of conditioned fear memory, such as contextual fear learning, as well as the acquisition and extinction of contextual fear conditioning. Lesion studies have revealed direct projections between the ventral hippocampus and both the infralimbic cortex and the basolateral amygdala (Hugues and Garcia, 2007), highlighting the crucial role of this region in modulating contextual fear responses (Gewirtz et al., 2000). Studies that demonstrate connections between the amygdala, the ventromedial prefrontal cortex (vmPFC), and the hippocampus further support the idea that the hippocampus plays a role in monitoring contextual fear conditioning (for a review see Maren et al., 2013).

## 3. Neurochemistry of kynurenine in the human brain

Kynurenine (KYN) is a metabolite of the amino acid tryptophan (Trp) that is utilized in the synthesis of nicotinamide adenine dinucleotide (NAD+). Physiologically, KYN is produced by the enzyme tryptophan dioxygenase (TDO), primarily in the liver, and indoleamine 2,3-dioxygenases (IDOs), which are synthesized in various tissues including the brain in response to immune-mediated activation (Opitz et al., 2011). KYN and its breakdown products serve multiple biological functions, such as blood vessel dilation during inflammation (Wang et al., 2010) and regulation of the immune response (Nguyen et al., 2010). KYN was initially identified in studies examining the chemical composition of canine urine (Battaglia et al., 2021), while its status as an intermediate metabolite of Trp was discovered half a century later (Musajo and Benassi, 1964). Through a series of endogenous reactions, a type of glial cell called astrocytes convert Trp into NAD+, a coenzyme that plays a vital role in cellular energy metabolism. Currently, the primary focus on Trp lies in its bioactive product serotonin (5-HT), which represents only 3% or less of Trp metabolism, while the KYN metabolic pathway accounts for approximately 90% (Stone and Darlington, 2002).

The metabolism of kynurenine (KYN) begins with the oxidation of the indole ring of tryptophan (Trp) by heme-containing enzymes, namely indoleamine 2,3-dioxygenases (IDOs) and tryptophan dioxygenase (TDO). This process leads to the production of N-formylkynurenine in the brain and other peripheral tissues. The activity of IDOs relies on superoxide (O2) and can be reduced from its inactive form (ferric) to its active form (ferrous) using reducing agents *in vitro*. Hence, it is suggested that IDOs could serve as antioxidant metabolites (Sono, 1986). The KYN metabolic pathway continues with the conversion of N-formylkynurenine to KYN by the enzyme formamidase, acting as a substrate for several enzymes. Under normal physiological conditions, kynureninase and kynurenine 3-hydroxylase convert KYN to anthranilic acid (AA) and 3-hydroxykynurenine (3-HK), respectively. AA is further converted to 3-hydroxyanthranilic acid (3-HAA) by the enzyme anthranilate 3-monooxygenase. Similarly, 3-HK is converted to 3-HAA by kynureninase. The enzyme 3-hydroxyanthranilate 3,4-dioxygenase (3-HAO) converts 3-HAA to 2-amino-3-carboxymuconate semialdehyde (ACMS). ACMS undergoes further conversion by the enzymes quinolinate phosphoribosyltransferase (QPRT) and iminoquinolinate dehydratase (IQD) to form quinolinic acid (QUIN). QUIN is then metabolized by a series of enzymes, including quinolinate phosphoribosyl transferase (QPRT), ultimately leading to the formation of NAD+. Immunohistochemical studies have shown that 3-HAO and QPRT are primarily present in the frontal neocortex, striatum, and hippocampus (Pérez-De La Cruz et al., 2007). Additionally, the remaining portion of KYN is irreversibly transaminated to kynurenic acid (KYNA) by the enzymes known as kynurenine aminotransferases (KATs).

Increasing attention has been given to kynurenine (KYN) not only from a chemical standpoint but also in the field of neurology, as it has implications in neuroinflammation and related immune responses. Recent evidence suggests that elevated KYN production may have significant implications in psychiatric conditions. For example, KYN has been associated with depressive symptoms in individuals undergoing interferon treatment for hepatitis C (Capuron et al., 2003). Cognitive deficits observed in schizophrenia have been attributed to enzymes involved in KYN breakdown (Wonodi et al., 2011) and reduced KYN levels have been found in the blood of patients with bipolar disorder (Bartoli et al., 2021). Furthermore, KYN synthesis is increased in Alzheimer's disease (Guillemin et al., 2005) and cardiovascular disease (Wirleitner et al., 2003), and its metabolites have been linked to cognitive deficits and depressive symptoms in these conditions (Swardfager et al., 2009; Gulaj et al., 2010); additionally, KYN appears to be associated with tics (McCreary and Handley, 1995; Hoekstra et al., 2007). Consequently, the KYN metabolic pathway has gained recognition for its association with inflammation, the immune system, and neurological disorders (Peters, 1991). Dysregulation or excessive activation of this pathway could lead to immune system responses and the accumulation of potentially neurotoxic compounds (Davis and Liu, 2015). Indeed, KYN deficiency has been linked to liver diseases (Hirata et al., 1967; Hoekstra et al., 2007; Holtze et al., 2012; Buness et al., 2014; Campbell et al., 2014) and brain disorders, including major depressive disorder, bipolar disorder, schizophrenia, and tic disorders (Marx et al., 2021).

Finally, within the human brain, various compositions of kynurenine aminotransferase (KAT) enzymes can be found, with KAT I and KAT II being the most significant ones. KAT II, in particular, is the primary enzyme involved in kynurenic acid (KYNA) synthesis. Both quinolinic acid (QUIN) and KYNA have been demonstrated to act as negative allosteric modulators of α7 nicotinic cholinergic receptors (α7nAChR), as antagonists at glutamate ionotropic receptors, and as antagonists of glutamate receptors such as NMDA receptors (NMDAR), amino-3-hydroxyl-5-methyl-4-isoxazole-propionate (AMPA) receptors, and kainite receptors, which play crucial roles in learning and memory processes (Robbins and Murphy, 2006). However, the specific *in vivo* actions of KYNA on α7nAChR are still being determined, and the effects of KYNA on aversive associative memory and antidepressant-like effects are influenced by factors such as concentration, microenvironments, and interactions with other neural circuits (Prescott et al., 2006; Rózsa et al., 2008; Tanaka et al., 2020a; Martos et al., 2022). Moreover, studies have shown that pharmacological inhibition of KAT II leads to a reduction in brain KYNA levels, effectively inhibiting de novo KYNA synthesis (Pocivavsek et al., 2019). This inhibition also prevents the stress-induced increase in KYNA observed in the prefrontal cortex (PFC) (Klausing et al., 2020), which appears to improve cognitive functions. Therefore, the modulation of kynurenine metabolism could be a targeted strategy for enhancing cognitive deficits and associated impairments in fear learning.

## 4. Kynurenine associated to learned fear

Fear learning experimental research has extensively investigated the crucial role of the essential amino acid tryptophan (Trp) in human fear-related phenomena. The kynurenine (KYN) metabolic route is a major candidate in the stress-activated inflammation pathway, particularly involved in the triggering of hyper-fearfulness. Increased expression of IDO1 and TDO2 enzymes, produced by immune cells and other cell types in different tissues, promotes Trp catabolism toward KYN in both the periphery and the brain (Mándi and Vécsei, 2012; Gibney et al., 2014; Nold et al., 2019; Tanaka and Vécsei, 2021; Tanaka et al., 2021a; Martos et al., 2022).

Although acute stress has been found to increase cerebral kynurenic acid (KYNA) levels in the fetus (Notarangelo and Pocivavsek, 2017) and in adulthood (Pawlak et al., 2000), research on human fear learning has primarily focused on the role of KYNA. KYNA is a metabolite derived from astrocytes that bi-directionally influences cognitive functions. Therefore, experimental increases in brain KYNA levels induced by systemic administration of KYN contribute to impairments in PFC-mediated set-shifting (Alexander et al., 2012), spatial contextual memory (Pocivavsek et al., 2011), fear learning (Chess et al., 2009), contextual fear learning (Akagbosu et al., 2012), and working memory (Chess et al., 2007).

**Figure 1:**
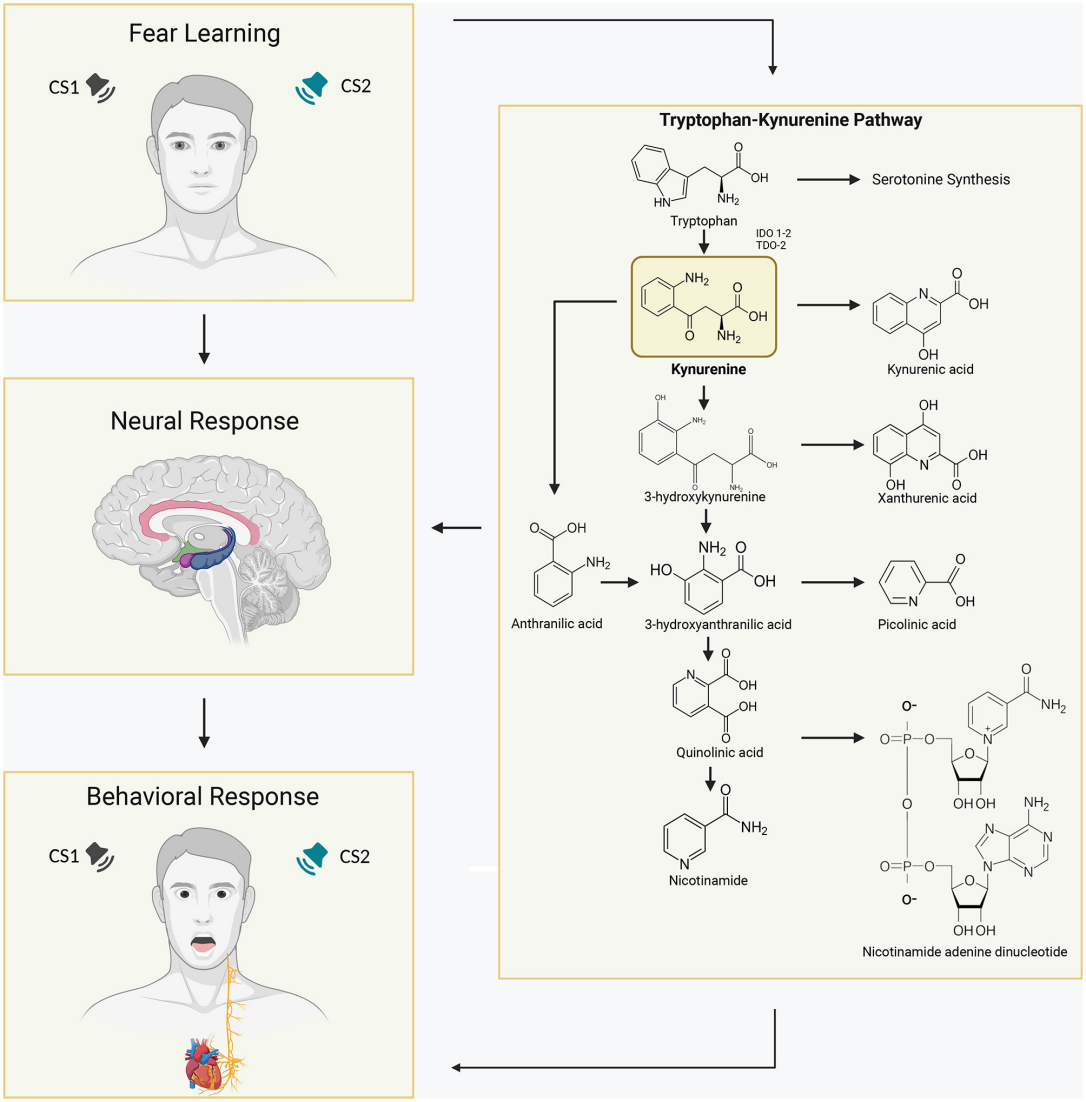
Schematic overview of the activated Tryptophan-Kynurenine metabolic system in the human brain and its association with learned fear. Tryptophan (Trp) is converted into kynurenine (KYN) by the enzymes indoleamine 2,3-dioxygenases (IDOs) and tryptophan dioxygenase (TDO). IDO-1 is expressed in various immune cells in the body, such as dendritic cells, monocytes, and macrophages, while IDO-2 is more selectively expressed in dendritic cells, liver, and kidney. KYN can be further metabolized into kynurenic acid (KYNA), which is typically considered to have neuroprotective properties, by the enzyme kynurenine aminotransferase (KAT). Alternatively, KYN can be converted into anthranilic acid by the enzyme kynureninase or into 3-hydroxykynurenine (3HK) by kynurenine mono-oxygenase (KMO). Consequently, elevated levels of brain KYNA have detrimental effects on various aspects of learning and memory, including fear learning. In fear learning paradigms, conditioned fear responses develop when a neutral stimulus is paired with an inherently aversive stimulus, and subsequent presentation of the conditioned stimulus alone elicits fear responses. Increased concentrations of the KYN metabolite kynurenic acid in specific regions of the brain involved in human fear learning, such as the amygdala and hippocampus, have been associated with altered and pathological fear states, influencing physiological and behavioral responses.

On the other hand, reduced levels of kynurenic acid (KYNA) achieved through pharmacological inhibition or genetic deletion of kynurenine aminotransferase II (KAT II), the main enzyme responsible for the synthesis of readily mobilizable KYNA in the mammalian brain, improve cognitive function (Kozak et al., 2014; Pocivavsek et al., 2019). These modulatory effects of endogenous KYNA are specifically associated with its interference with the function of α7 nicotinic cholinergic receptors (α7nAChR) and N-methyl-D-aspartate receptors (NMDARs), both of which play crucial roles in learning and memory (Hilmas et al., 2001; Robbins and Murphy, 2006). Therefore, the elevated levels of KYNA observed in fear learning and memory are believed to primarily affect NMDA receptors in critical regions for human fear learning, such as the amygdala and hippocampus (Chess et al., 2007, 2009). This supports the notion that a physiologically relevant increase in KYNA concentration has a significant impact on NMDA receptors. Evidence suggests a general deficit in contextual learning and discrimination when KYNA levels are elevated, indicating that it slows down contextual discrimination rather than preventing it. This effect may be related to dysfunctional cue-based behaviors and sensory processing (Wu et al., 2000; Figure 1).

Moreover, individuals with schizophrenia (SCZ) and bipolar disorder exhibit elevated concentrations of kynurenic acid (KYNA) in their cerebrospinal fluid (CSF) and cortex (Akagbosu et al., 2012; Iaccarino et al., 2013; Fuertig et al., 2016; Pershing et al., 2016). Schizophrenic patients have approximately 1.5 times higher levels of kynurenine and KYNA in the brain, while the concentrations of kynurenine and KYNA in the CSF are approximately 2 and 1.5 times higher, respectively, in SCZ patients compared to healthy controls. Similarly, patients with bipolar disorder show a 1.5 times increase in KYNA levels in their CSF (Holtze et al., 2012; Linderholm et al., 2012). Haplotype analysis has revealed that gene polymorphisms in the kynurenine 3-monooxygenase (KMO) gene are associated with KYNA concentration in the CSF of SCZ patients. KMO is responsible for the initial breakdown of kynurenine, and importantly, patients with SCZ and bipolar disorder demonstrate lower levels of KMO mRNA (Lavebratt et al., 2014).

## 5. Conclusion and future perspective

Higher levels of the KYN metabolite kynurenic acid (KYNA) in the human brain have been associated with altered fear states resulting from trauma, stress, and anxiety (Erhardt et al., 2017a,b; Borgomaneri et al., 2021a,b; Di Gregorio et al., 2021; Tanaka and Vécsei, 2021; Battaglia, 2022; Di Gregorio and Battaglia, 2023). These elevated KYNA levels may contribute to the cognitive and sensory deficits observed in these disorders (Erhardt et al., 2007; Amori et al., 2009; Athnaiel et al., 2022; Battaglia et al., 2022a,b; Tanaka et al., 2023). KYNA concentrations are found to be increased in areas such as the prefrontal cortex (PFC) and cerebrospinal fluid (CSF) of patients with psychiatric disorders (Erhardt et al., 2001; Nilsson et al., 2005, 2007; Linderholm et al., 2012; Tanaka et al., 2020b, 2021a). Moreover, experimental manipulation of brain KYNA levels through systemic administration of KYN has been shown to result in impairments in PFC-mediated set-shifting (Alexander et al., 2012), spatial contextual memory, fear learning (Chess et al., 2009), and working memory capacities (Chess et al., 2007). In contrast, reducing cerebral KYNA levels through pharmacological inhibition or genetic deletion of KAT II, the primary enzyme responsible for KYNA production in the human brain, has been found to improve cognitive functions (Kozak et al., 2014; Pocivavsek et al., 2019).

Finally, this work enhances our understanding of the metabolic substrates that establish a causal connection between increased KYNA levels and alterations in PFC-dependent fear behavior. While current research has mostly focused on the neuroprotective aspects of KYN production, our focus has been on the targeted regulation of this downstream metabolism and its implications for neuropathology and ehavioural disorders. Therefore, future perspectives aim to test the hypothesis that PFC-dependent behaviors may be particularly susceptible to neurotoxic dysregulation of KYN metabolism, which, in turn, could have significant implications for the diagnosis and potential treatment of neuropsychiatric and neurodegenerative disorders.

## Author contributions

SB: conceptualization, supervision, project administration, and funding acquisition. MB, CD, and SB: writing—original draft, review, and editing. SB and CD: visualization. All authors have read and agreed to the published version of the manuscript.
